# Early alterations in retinal microvasculature on swept-source optical coherence tomography angiography in acute central serous chorioretinopathy

**DOI:** 10.1038/s41598-021-82650-1

**Published:** 2021-02-04

**Authors:** Dominika Podkowinski, Bernhard Foessl, Luis de Sisternes, Sophie Beka, Anna-Sophie Mursch-Edlmayr, Rupert W. Strauss, Matthias Bolz

**Affiliations:** 1 Department of Ophthalmology, Kepler University Clinic, Linz, Austria; 2 Johannes Kepler University, Linz, Austria; 3 Carl Zeiss Meditec Inc., Dublin, CA USA; 4 Department of Ophthalmology, Medical University Graz, Graz, Austria; 5 Department of Ophthalmology and Optometry, Kepler University Clinic, Med Campus III, Krankenhausstrasse 9, 4021 Linz, Austria

**Keywords:** Eye diseases, Retinal diseases

## Abstract

The purpose of the study was to evaluate the retinal blood flow in patients with acute central serous chorioretinopathy (CSC) over an observational period of 1 month using swept-source optical coherence tomography (SS-OCTA), focusing especially on changes in the area of subretinal fluid (A-SRF). We correlated these findings with conventional indocyanine green angiography (ICGA). ICGA and SS-OCTA images were collected and analyzed of 12 eyes of 12 patients. The A-SRF was annotated and a qualitative analysis of choriocapillaris, the vessel density (VD) and perfusion density (PD) of the retinal superficial capillary plexus (SCP) and the deep capillary plexus (DCP) was performed in A-SRF and the unaffected remaining area (RA). The VD and PD in the DCP were statistically significantly lower in A-SRF than in the RA at baseline. (VD: *p* = 0.014; PD: *p* = 0.036). After 1 month, there was a statistically significant difference in the VD and PD of the DCP (VD: *p* = 0.015; PD: *p* = 0.014), and for the PD of the SCP between the A-SRF and the RA (*p* = 0.015), with lower values in the A-SRF. We found low perfused areas in choriocapillaris corresponding to hypofluorescent areas on ICGA. In conclusion there is a difference in VD and VD of the DCP in the area of SRF in acute CSC. These alterations may lead to a chronic change in the microvasculature and potentially to morphological changes**.**

## Introduction

Central serous chorioretinopathy (CSC) is a disease of variable duration affecting predominantly middle-aged patients and is characterized by serous retinal detachment of the macula or at the posterior pole^1,2^. This may result in visual impairment, especially in patients with a chronic disease course, due to outer retinal atrophy or secondarily to choroidal neovascularization^3^. However, acute CSC is mostly a self-limiting disease with a satisfactory visual prognosis^4^. Differentiation between acute and chronic CSC is based on duration of symptoms due to the presence of subretinal fluid (SRF) and morphological findings on optical coherence tomography (OCT) scans, as well as in fluorescein angiography (FA) and indocyanine green angiography (ICGA) imaging^5–7^. FA images provide typical angiographic changes in CSC, but may fail to visualize possible choroidal pathologies, as the dye is absorbed by the retinal pigment epithelium (RPE). ICGA shows areas of choroidal leakage attributable to hyperpermeability of the choriocapillaris that leads consequently to the accumulation of subretinal fluid^8^. This separation of the photoreceptors from the choroid and RPE can cause hypoxia of the outer retina^9^.

With the novel image technology of swept-source optical coherence tomography angiography (SS-OCTA), a new non-invasive approach of evaluating retinal blood flow and choroidal neovascularization in retinal diseases is available^10,11^. As the pathophysiology of CSC is so far not completely understood, SS-OCTA may provide novel insights into the disease pathophysiology and its natural course.

Reports about the use of OCTA in CSC are scant, but OCTA revealed changes in retinal capillary vessels, as well as irregular choriocapillaris flow patterns in chronic CSC patients^12,13^. A study performed by Feucht et al*.* analyzing patients at a single time point couldn’t detect any retinal microvascular changes, although the authors were able to visualize an altered choroidal flow in a cross-sectional analysis^14^. To the best of our knowledge, there are no longitudinal reports of retinal microvasculature in patients with CSC, especially in patients with acute CSC.

Hu et al. demonstrated three types of choriocapillaris changes in OCTA^15^. In acute and chronic CSC, Type A is defined as a coarse-granulated high-reflective area, Type B as a dark halo around Type A and Type C as a coarse-granulated low-reflective area inside Type A.

So far there is no study evaluating the possible short-time changes and longitudinal effects in retinal blood flow in patients with acute CSC using OCTA. Therefore we investigated the retinal microvasculature in patients with acute CSC over time using standard imaging tools (OCT and FA/ICGA), and to compare it with SS-OCTA. The aim of the study was to analyze the region of subretinal fluid and it’s microvasculature changes in detail. As subretinal fluid is one of the key clinical features in patients with acute CSC this area is of great interest. To the best of our knowledge this is the first study to evaluate changes in blood flow in acute CSC by using SS-OCTA over a short period of time.

## Results

Twelve eyes (five right, seven left) of 12 patients (nine males and three females) were included in this study. The mean age of the patients at first (baseline) visit was 48.3 ± 11.4 years. Two patients had diabetes mellitus type two (no sign of diabetic retinopathy), one patient had psoriasis vulgaris and one patient was diagnosed with polyarthritis. Three patients were diagnosed with clinical depression prior to our study. Six patients reported subjective stressful periods prior to the occurrence of the CSC. Only one patient used a topical cortisol cream, none of the other patients reported prior intake or use of steroid medication. Apart of one patient none of the patients had hypertension. Three patients were smokers.

In all cases, the subretinal fluid was located within and completely covered by the ETDRS grid. All annotated SRF areas were in the 6 mm ETDRS grid. The area of the SRF at baseline was 5.0 ± 5.0 mm^2^ and 3.2 ± 4.7 mm^2^ at month one (*p* = 0.182). However, one patient showed resolution of the SRF at month one.

At baseline there were no statistically significant differences in vessel density or perfusion density of the SCP between the A-SRF compared to the remaining reference area (RA). At month one there was statistically significant difference in the perfusion density in the SCP with lower values in the A-SRF. The vessel density in the SCP was similar between the A-SRF and the RA. The mean vessel and perfusion density in the DCP at baseline and month one was statistically significantly lower in the area of the A-SRF than in the 6 mm area RA. All values are displayed in Table 1.

**Table 1:** Vessel perfusion density analysis for subretinal fluid and ETDRS grid (6 mm).

	SRF	ETDRS grid without SRF	*p*
**Baseline**			
N =	12	12	
Vessel density SCP (mean ± SD)	17.4 ± 2.2	18.6 ± 2.7	0.149
Vessel density DCP (mean ± SD)	12.1 ± 3.9	16.1 ± 2.7	**0.014**
Perfusion density SCP (mean ± SD)	0.4 ± 0.06	0.44 ± 0.04	0.203
Perfusion density DCP (mean ± SD)	0.26 ± 0.08	0.34 ± 0.06	**0.036**
**Month 1**			
N =	11	11	
Vessel density SCP (mean ± SD)	16.7 ± 4.1	19.5 ± 1.5	0.074
Vessel density DCP (mean ± SD)	10.3 ± 6.8	16.2 ± 3.1	**0.015**
Perfusion density SCP (mean ± SD)	0.35 ± 0.09	0.45 ± 0.03	**0.014**
Perfusion density DCP (mean ± SD)	0.21 ± 0.14	0.34 ± 0.06	**0.015**

Stastistically signficant *p* values are marked in bold.

*SRF* subretinal fluid, *ETDRS* early treatment of diabetic retinopathy study, *mm* millimetre, *SCP* superficial capillary plexus, *DCP* deep capillary plexus, *SD* standard deviation.

The vessel density (mean ± standard deviation) of the SCP (baseline: 16.3 ± 4.3, month 1: 16.7 ± 4.1; *p* = 0.72) and DCP (baseline: 12.3 ± 4.2, month 1: 11.4 ± 6.2; *p* = 0.36) and the perfusion density of the SCP (baseline: 0.37 ± 0.11; month 1: 0.35 ± 0.09; *p* = 0.40) and DCP (baseline: 0.23 ± 0.11, month 1: 0.21 ± 0.15; *p* = 0.40) of the A-SRF were not statistically significant between baseline and after 1 month. None of the patients developed secondary choroidal neovascularizations neither on FA/ICGA nor on SS-OCTA images. The vessel density of the SCP of area with resolved SRF at month 1 compared to the A-SRF at month 1 was statistically significantly higher (*p* = 0.015). The perfusion density in the SCP, the vessel and perfusion density in the DCP was not statistically different (*p* > 0.05). Values are displayed in Table 2.

**Table 2:** Vessel and perfusion density analysis for subretinal fluid and areas with resolved SRF at month one.

Baseline	SRF	Resolved SRF area	*p*
N =	11	11	
Vessel density SCP (mean ± SD)	16.7 ± 4.1	17.6 ± 4.7	**0.013**
Vessel density DCP (mean ± SD)	11.4 ± 6.3	13.6 ± 4.6	0.063
Perfusion density SCP (mean ± SD)	0.35 ± 0.09	0.44 ± 0.03	0.490
Perfusion density DCP (mean ± SD)	0.23 ± 0.14	0.35 ± 0.09	0.070

Stastistically signficant *p* values are marked in bold.

*SRF* subretinal fluid, *mm* millimetre, *SCP* superficial capillary plexus, *DCP* deep capillary plexus, *SD* standard deviation.

### Qualitative analysis of the choriocapillaris

**Figure 1:**
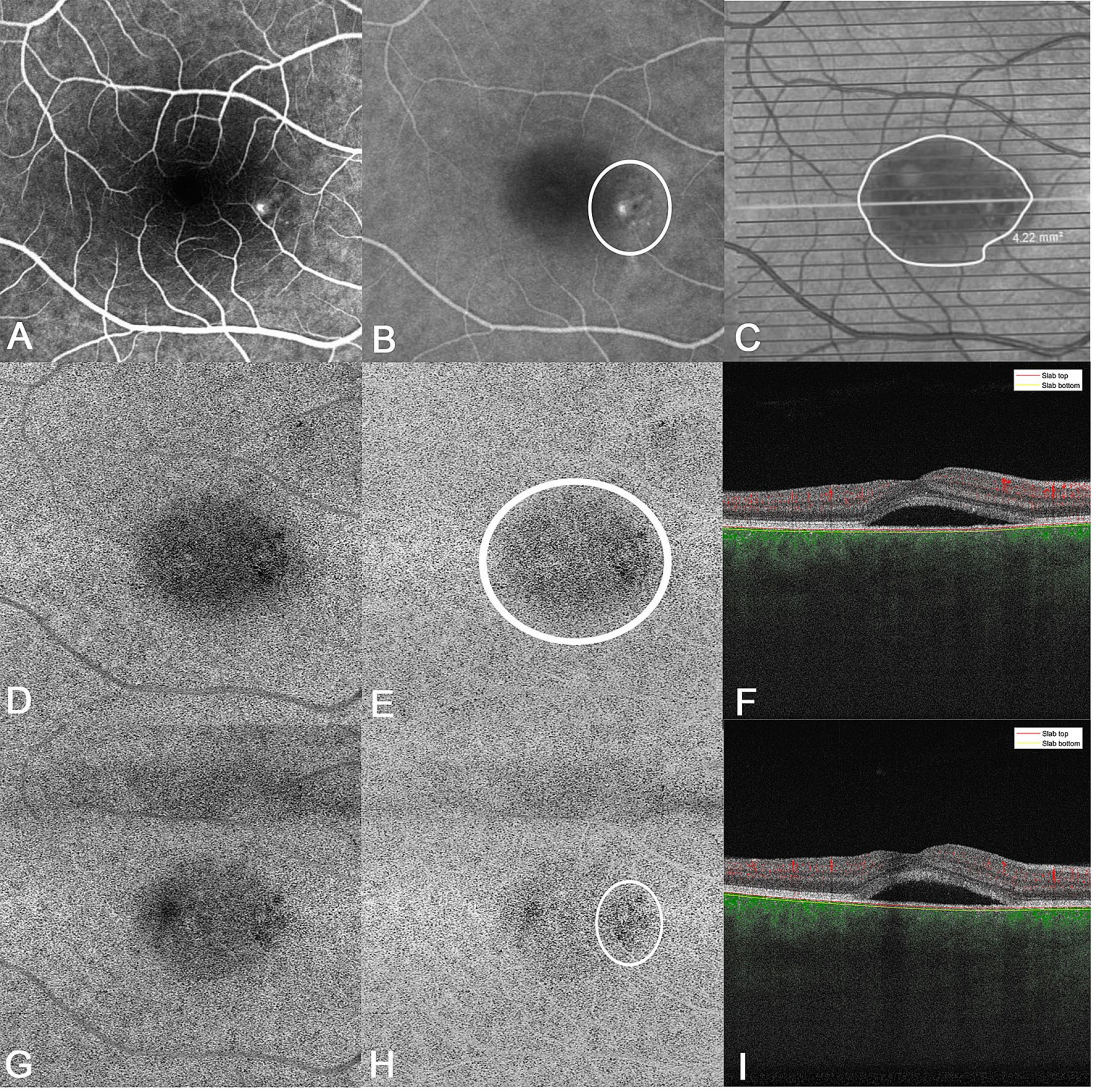
Example of choriocapillaris analysis. (**A**) Fluorescein angiogram showing leakage point, (**B**) indocyanin green angiogram (ICGA) showing hypofluorescent areas and leakage point marked with the white circle, (**C**) en face OCT image with the marked SRF area, (**D**) angiography image of the choriocapillaris slab at baseline prior to compensation and projection removal. Projection artifact of the SRF is visible. (**E**) Angiography image of the choriocapillaris slab at baseline after compensation and projection removal. There is a diffuse gray area visible (white circle), (**F**) central B-scan at baseline showing SRF. The slabs mark the segmentation lines of the choriocapillaris, (**G**) angiography image of the choriocapillaris slab at month one prior to compensation and projection removal. Projection artifact of the SRF is visible. (**H**) Angiography image of the choriocapillaris slab at month one after compensation and projection removal. The gray area is smaller compared to baseline, although SRF is still present. The white circle marks lower perfusion in the choriocapillaris, corresponding area of the leakage point and hypoflourescence on ICGA (image B). (**I**) Central B-scan at month one showing SRF. The slabs mark the segmentation lines of the choriocapillaris.

Prior to the compensation and projection removal of the retinal vessels, there was a clear shadowing visible from the SRF (Fig. 1). After image processing, all of the patients showed diffuse gray areas in the choriocapillaris at baseline. The gray areas visible on SS-OCTA were consistent with hypofluorescent areas on ICGA images. These areas were less diffuse at follow-up of 1 month and showed localized dark spots in the CC, which were localized around the vessel leakage on FA/ICGA images or were congruent with hypofluorescent areas on ICGA.

Eight patients showed Type C changes on baseline, but without the surrounding Type A changes. Two patients showed Type C changes with surrounding Type A changes. One patient showed solely Type A, and one patient only Type B changes at baseline.

There were no Type B changes at month one. The patients with Type B changes at baseline showed Type C with surrounding Type A changes. All the other patients showed the same changes at baseline as at month one.

## Discussion

In this study, we investigated the retinal perfusion and analyzed choriocapillaris patterns in patients with acute CSC. We detected lower perfusion and vessel density in the deep capillary plexus in the area of the subretinal fluid compared to the surrounding region within the 6 mm EDTRS grid centered on the fovea. The analysis of the choriocapillaris revealed flow irregularities in the area around the leakage point as determined by FA imaging, which may be interpreted as an early damage to the choriocapillaris in patients with acute CSC.

CSC is known to be a disease originating in the choroid that leads to subretinal fluid. When using FA imaging in acute CSC patients there is a typical phenomenon of a staining point with otherwise normal retinal perfusion. However, FA imaging is a two-dimensional technique and differentiation between capillary layers is not possible, whereas SS-OCTA imaging can provide valuable insight into the perfusion status of the retina and choroid.

A study comparing the density of the superficial microvasculature using OCTA with controls found a decreased density in patients with CSC^16^. However, other authors (examining a larger cohort) were not able to reproduce these results in the superficial plexus for acute CSC patients, but found a decreased density and perfusion for chronic CSC^12^. This difference may depend on the different time points chosen in the different studies, as we were able to demonstrate a decrease in vessel density of the SCP after 1 month in the SRF area and not at baseline, meaning symptoms described by the patients were less than a month. Therefore it may be conceivable that changes in SCP appear after longer persistence of SRF. Additionally, prior studies had a cross-sectional design and changes over time can’t be shown. Our study is the first longitudinal report on retinal microvasculature in patients with acute CSC.

The division of the capillary plexus employed in this study simply differentiating between SCP and DCP may not be perfect, as the deep capillary plexus can be seen as part of a deep vascular complex (DVP) with the intermediate capillary plexus^17^. However, for the purpose of study it was feasible to differentiate only between the SCP and the DCP as the SCP supplies the inner retinal layers, whereas the DCP provides perfusion and oxygen supply to the photoreceptor axon terminals localized in the outer nuclear layer, and therefore is crucial for the function of the photoreceptors. In our study, we found decreased density and perfusion density in the area of SRF, showing an early manifested change in retinal blood flow in patients with acute CSC. This may lead to a persistent damage of the photoreceptors and furthermore poorer visual outcomes. A recent study analyzing retinal perfusion after low-fluence photodynamic therapy (PDT) demonstrated an increase in vessel density in the DCP in patients with chronic CSC^18^. The authors hypothesize that the increase in vessel density is caused by the reduction of subretinal fluid and normalization of the central retinal thickness. In contrast, we were not able to detect a significant change in perfusion or density in the area of previous SRF after 1 month in the DCP. Therefore the disappearance of SRF does not fully explain a density increase in the DCP. It is conceivable, that there is still a disturbed or dysfunctional retinal blood flow. A recent study using flicker light stimulation showed a decreased retinal vein response to flicker light stimulation leading to a venous stasis^19^. This may lead to a decreased capillary perfusion, which would support our findings. This would be supported by the theory, that CSC is primarily based on RPE dysfunction. Ion channels in the RPE cells are disrupted due to RPE damage leading to an outer retinal blood breakdown^20^. The fluid movement is reversed in a chorioretinal direction. It may be conceivable that this gradient impacts the retinal blood flow in the DCP especially at the SRF area, as demonstrated in our results. Further studies will be necessary to explore if the changes in retinal microvasculature persist after absorption of SRF. It has to be noted that the presented study has a short follow-up time of 1 month. Long-term changes or self-regulatory mechanism may be difficult to grasp.

Choriocapillaris alterations have not only been found in CSC patients, but also in patients with other retinal diseases or in myopic eyes^21,22^. A study analyzing the choriocapillaris with OCTA found that low flow in the choriocapillaris puts neighboring areas at risk of further flow impairment^23^. This would be in alignment with our results of a defined area of low perfused choriocapillaris around the leakage point-suggesting acute choriocapillaris damage with affection of the surrounding areas. In our study we used scans of 6 × 6 mm field of view, which can provide a wider view of the choriocapillaris, compared to scans of 3 × 3 mm used in the study by Spaide^23^*.* Furthermore, the laser of SS-OCTA devices penetrates deeper and therefore the choriocapillaris presentation in our work may be of higher quality compared to previous studies using spectral domain OCTA.

Another study defined “dark spots” and “dark areas” in the choriocapillaris using SD-OCTA in patients with CSC^24^. The authors concluded that these dark areas could be caused by artifacts due to PED or SRF, or be caused by flow reductions in the capillary layers. A recent study by Xu et al*.* showed an effect of PDT on the irregular choriocapillaris flow patterns, which existed prior to the treatment^25^. Authors therefore suggest that PDT may restore the capillary flow. However, when analyzing our data, we have seen an overall apparent significant reduction of the choriocapillaris flow within the SRF region. Therefore, we hypothesize that the improved flow within the SRF region may just be artifactual and an effect of the resolved SRF after treatment. After removing the artifacts on the choriocapillaris caused by SRF, still a more localized reduction in flow in the choriocapillaris was visible at the SRF at baseline and 1 month. Therefore it is very likely that there is a real reduction in flow in the choriocapillaris within these regions localized around the leakage point visible in FA and ICGA images. As our patients showed symptoms for less than 1 month there must be an early damage in the choriocapillaris, which may precede the appearance of the SRF. This would support one theory of the origin of CSC: the primary starting point is at the level of the choriocapillaris leading to dysfunction of the RPE and finally to the SRF^26^. Doppler flowmetry demonstrated lower blood flow at the level of the choriocapillaris in patients with CSC compared to healthy controls^27^. This in contrast to the above mentioned theory of primary RPE dysfunction and it still remains unclear, which theory applies to this finding.

In another study high-intensity lesions were found in the choriocapillaris associated with the leakage area on FA images^28^. We did not see these high-intensity lesions in our patients, which could be due to short duration of the disease. It may be conceivable that these high intensity signals occur as a compensatory mechanism further on in the disease. *Nicolo *et al*.* hypothesize, that there may be a vasoconstriction of the choriocapillaris to compensate for the restricted autoregulatory mechanisms of the choroid^29^. They showed a larger flow area of the choroidal vessels in the CSC patients compared to healthy controls. These findings are in alignment with prior OCT studies showing increased choroidal vascularity and thickened choroids compared to healthy eyes^30,31^. Hence the focus in our study was on the choriocapillaris, as choroidal alterations are already well described in the literature.

In our study Type B changes caused by the shadow effect were visible only prior to the projection and compensation step, shown in Fig. 1d,e,g,h, except one patient where the Type B changes were visible after compensation at baseline. Therefore it is likely that Type B, as described by the authors, is an artifact caused by the presence of SRF. Therefore, Type B changes may be found more often in acute CSC. Furthermore, in most of the Type C changes we were not able to see the surrounding granulated high-reflective area (Type A). As our study is the first study to analyze these choriocapillaris alterations using SS-OCTA technology, the discrepancies between our results and the study conducted by Hu et al. are explicable. The authors speculated that in may be caused by the dilation and increased blood flow of choriocapillaris, but provided no further explanation. On the one hand the compensation process, used in our study, may cause the differences. On the other hand it could be due to the better visualization using the swept-source technology. Therefore our results highlight the importance of correct OCTA image analysis, as artifacts and segmentation are well-known confounding factors in OCTA^32^. Standardized grading protocols are useful tools to compare study results in daily-clinical practise. It still remains unclear how the choriocapillaris changes may be impacted by possible treatment. Establishing choriocapillaris phenotypes may enable the differentiation of CSC subgroups and develop personalized treatment strategies.

Although acute CSC is mostly a self-limiting disease, recurrence rates in the literature are reported up to 50%^33^. Such early changes in choriocapillaris and changes in retinal microvasculature in the SRF area may be part of the underlying cause for recurrence of CSC and further poorer visual acuity outcomes.

There are multiple known risk factors for CSC. In our study half of the participants described stressful periods prior to diagnosis of CSC. However it may be difficult to access stress objectively even if using self-reporting questionnaires^26^. 25% of our patients had clinically diagnosed depression with is another risk factor described in the literature and it was described that the severity of the depression may even correlate with the size of choroidal changes^34^. Interestingly only one patient used corticosteroids, in the form of a facial cream, prior to this study. As the use corticosteroids is a well-established risk factor it is interesting, that the number is so low. However recent reports suggest that there is a clear role of the mineralocorticoid receptor and glucocorticoids in patients with CSC^26^.

The main limitation of this study is the small number of patients analyzed. However, because CSC is a rather rare disease, larger sample sizes are hard to collect. The strength of our study however is that our patients present a homogenous group of cases with acute CSC and complete imaging data at baseline and follow-up of 1 month. Further studies are necessary to access the long-term effects of the disease on retinal vasculature and the choriocapillaris. It cannot be excluded that some changes in perfusion or vessel density on OCTA may be due to projection and shadowing artifacts; however, the application of thorough images post-processing reduces this possible confounding factor significantly. Furthermore, a recent study showed reduced flow of the choriocapillaris even after the resolution of the SRF^35^.

In conclusion, we demonstrated the changes in retinal perfusion in patients with acute CSC using a SS-OCTA device. The perfusion and vessel density in the deep capillary were statistically lower in the deep capillary plexus within the area of the SRF at baseline and after 1 month showing an early change in retinal microvasculature. Changes in the superficial capillary plexus in the area of the SRF found at 1 month suggest that the duration of the SRF impacts the superficial capillary plexus. The choriocapillaris on SS-OCTA showed changes in perfusion at the leakage point. It can be hypothesized that even in the acute phase damages in perfusion on the level of choriocapillaris are present. This may have an impact on outcome and treatment of patients with acute CSC. Further studies are needed to investigate this effect in the microvasculature visible with OCTA imaging.

## Methods

Patients with the diagnosis of acute central serous chorioretinopathy, who presented in 2017 and 2018 to the outpatient clinic at the Department of Ophthalmology, Kepler University Clinic Linz (Linz, Austria) were included in this retrospective analysis. The study was performed according to the tenets of the Declaration of Helsinki. All participants provided written informed consent prior to enrollment. The study was approved by the Ethics Committee of the Federal State Upper Austria.

### Inclusion/exclusion criteria

Inclusion criteria were the presence of acute CSC based on the following criteria: (1) neurosensory detachment of the retina as determined by OCT; (2) fluorescein leakage points in FA and/or hyperfluorescent areas in ICGA; (3) new onset of disease, symptoms less than 1 month, without prior history of former CSC. FA, ICGA, and SS-OCTA images were performed on the same day at first visit (baseline). Exclusion criteria were: (1) any other retinal diseases and glaucoma; (2) previous history of CSC or ongoing treatment during the follow-up between baseline and month one for CSC (3) prior intraocular surgery; (4) presence of secondary choroidal neovascularization.

### Ophthalmological examination and imaging

All patients underwent at baseline an ophthalmological examination including best corrected visual acuity (BCVA), and detailed slit-lamp biomicroscopy including pupil dilatation and fundoscopy. Standardized OCT measurements were performed using the Spectralis HRA + OCT system (Heidelberg Engineering, Heidelberg, Germany). A 49-line raster OCT scan of 20° × 20° with 20 frames per second was performed followed by simultaneous fluorescein angiography (FA) and indocyanine green angiography (ICGA) to visualize vessel leakage. FA and ICGA imaging were performed at baseline only. Additionally SS-OCTA images were acquired using the SS-OCTA Plex Elite 900 (Zeiss Meditec, Dublin, California, USA). After 1 month, the patient was re-examined clinically and by OCT and SS-OCTA.

### Image analysis

**Figure 2:**
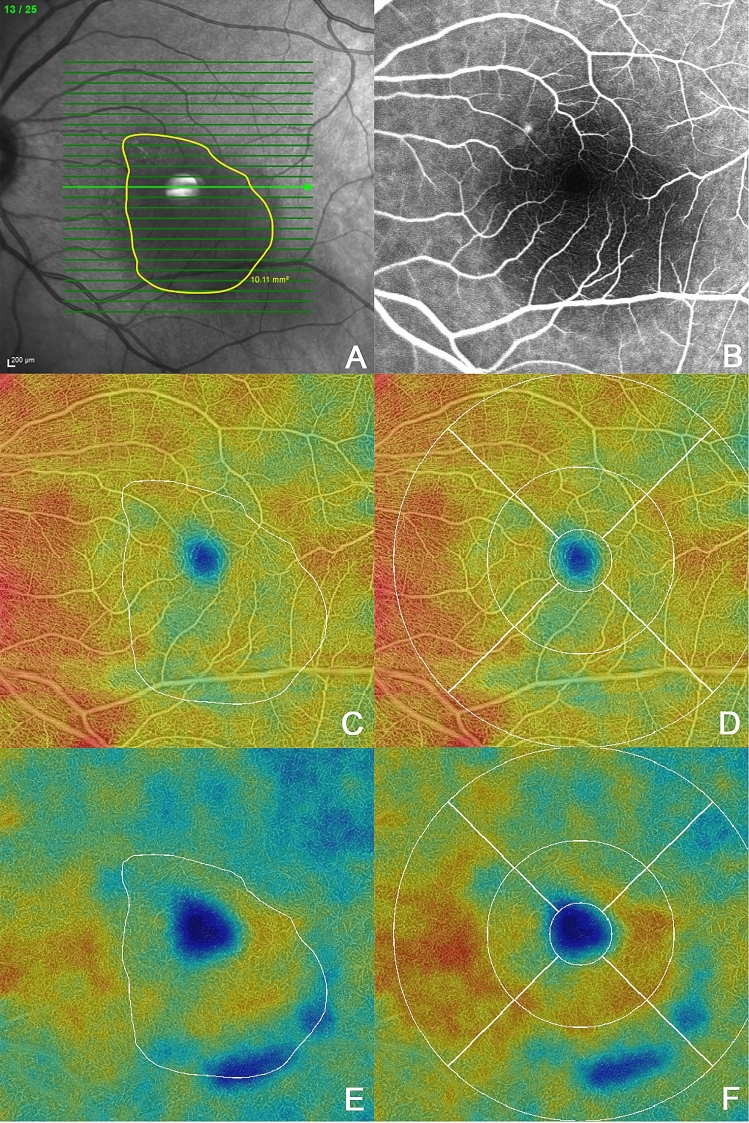
Example of vessel and perfusion density analysis. (**A**) En face image with annotated subretinal fluid (SRF) (**B**) Fluorescein angiogram at baseline showing a staining spot. (**C**) Marked SRF area on color-coded perfusion density map of the superficial capillary plexus (SCP) (**D**) 6 mm circle on color-coded perfusion density map of the SCP (**E**) Marked SRF area on color-coded perfusion density map of the deep capillary plexus (DCP) (**F**) 6 mm circle on color-coded perfusion density map of the DCP.

Images derived from SS-OCTA, FA, ICGA images were exported, registered and overlaid manually by an experienced reader (B.F.), using vessel crossing as anatomical landmarks. The subretinal fluid (SRF) was annotated in the overlay en-face images using ICG and FA angiography images to identify the leakage point(s). The area with SRF was defined as the area of interest (A-SRF). Furthermore, we overlaid the resulting annotations with their corresponding OCTA slabs (en face images obtained by projecting the flow information restricted to a particular depth within the OCTA volumetric acquisition, relative to a specific retina plexus). We used the inbuilt segmentation OCTA software to divide the OCTA volumes into superficial capillary plexus (SCP), deep capillary plexus (DCP), choroid, and choriocapillaris; producing the slabs of interest. Segmentation was corrected manually if segmentation errors occurred due to the presence of SRF. The exported SS-OCTA images were analyzed on the ARI Network platform using a prototype algorithm “Macular Density v0.7” provided by Carl Zeiss Meditec. We analyzed the vessel density and perfusion density for the SCP and DCP in the marked A-SRF and leakage area at baseline and month one for each patient. An example is demonstrated in Fig. 2. All images were registered between baseline and month 1 to perform comparison of the density metrics within the same regions. To analyze the possibility of decrease of these metrics within the A-SRF and leakage regions vs. surrounding regions we also compared the vessel density and perfusion in the A-SRF, at the leakage area with the area outside the A-SRF, corresponding to the remaining area (RA) covered by the ETDRS grid, which served as the reference area at baseline and after 1 month.

### Qualitative analysis of the choriocapillaris

In order to qualitatively assess the choriocapillaris (CC), we generated CC slab images following a similar approach as previously presented by Zhang et al. In the first step, motion artifacts were removed, followed by a removal of projection artifacts due to overlaying the retinal vessels^36^. In the second step, images were compensated to remove shadowing on the choriocapillaris due to subretinal fluid. We additionally used the type of choriocapillaris changes defined by Hu et al*.* for choriocapillaris grading^15^.

### Statistical analysis

The SPSS Statistics version 26 of software program (IBM Corp., Armonk, NY, USA) was used for statistical analysis. All of the analyzed parameters were checked for normality (Shapiro–Wilk, *p* > 0.05; Q–Q-Plot). For normally distributed variables a Student’s t-test was used to test for statistical differences between groups, for not normally distributed variables a Mann–Whitney U test was performed. The statistical significance level was set at *p* ≤ 0.05.

## Data Availability

The analyzed dataset is available from the corresponding author on reasonable request (email: matthias.bolz@kepleruniklinikum.at).
